# Ten-Year Cardiovascular Risk as Predicted by the QRISK®3 Calculator in Diabetic Patients Attending a Tertiary Care Teaching Hospital in Central India and Its Application to Stratify Statin Over-Users and Under-Users

**DOI:** 10.7759/cureus.47213

**Published:** 2023-10-17

**Authors:** Varnan Chandrawanshi, Nitin R Gaikwad, Yogendra Keche, Preetam Wasnik, Suryaprakash Dhaneria

**Affiliations:** 1 General Medicine, Mahatma Gandhi Memorial Medical College, Indore, IND; 2 Pharmacology, All India Institute of Medical Sciences, Raipur, IND; 3 General Medicine, All India Institute of Medical Sciences, Raipur, IND; 4 Pharmacology and Therapeutics, Ruxmaniben Deepchand Gardi Medical College, Ujjain, IND

**Keywords:** cardiovascular disease, qrisk, indian ethnicity, nice guidelines, statins, diabetes, cvd risk

## Abstract

Background: Cardiovascular disease (CVD) is an important cause of morbidity and mortality in diabetic patients. As such, risk stratification is essential to identify the risk factors of CVD and provide early intervention. The QRISK^®^3 tool, recommended by the National Institute for Health and Care Excellence (NICE) guidelines, has the option to choose the patient’s ethnicity, which is not available in other tools. However, there is a paucity of data regarding the use of this tool in the Indian population. Therefore, this study was planned to predict 10-year CVD risk using the QRISK^®^3 tool and to determine statin eligibility in diabetic patients.

Methods: We enrolled diabetic patients visiting our general medicine outpatient department and diabetic clinic in the study. We collected data from clinical and prescription records, as well as through patient interviews. We analyzed the data to determine the 10-year CVD risk using the QRISK^®^3 risk tool, which is available online. A cut-off QRISK score of 10%, as recommended by the NICE guidelines (2014), was used to stratify patients as “over-users” and “under-users.” We also analyzed the data to determine any correlation between other risk factors and QRISK scores.

Results: Of the 134 diabetic patients recruited in this study, 43 (32.09%) had a CVD risk score of <10%, of which 16 (37.21%) were categorized as “over-users.” Of the patients, 91 had a CVD risk score of ≥10%, of which 17 (18.68%) were categorized as “under-users.” Risk factors showing a positive correlation with QRISK score included duration of diabetes, age, blood pressure treatment, waist circumference, and non-high-density lipoprotein cholesterol level.

Conclusion: QRISK score can be useful to predict 10-year CVD risk in the Indian population and to stratify patients as statin over-users and under-users. This tool can be used in the Indian set-up to identify potential candidates for statin initiation.

## Introduction

Diabetes mellitus (DM) is a group of metabolic diseases [1]. The WHO Global Report 2016 estimated a global DM prevalence of 8.5%, with 69.2 million cases in India, placing it among the top three countries with the largest diabetic population [2]. An Indian study conducted by the Indian Council of Medical Research in 15 states reported an overall DM prevalence of 7.3% [3].

Cardiovascular disease (CVD) is a major complication of DM and the leading cause of diabetes-related morbidity and mortality [4]. Adults with DM have a two-to-three-fold increased risk of CVD compared with those without diabetes [5]. The risk factors for CVD include hypertension, diabetes, smoking, obesity, tobacco/alcohol use, physical inactivity, poor diet, psychosocial stress, low fruit and vegetable intake, and dyslipidemia [6].

Primary prevention of CVD in terms of risk stratification is essential to identify its risk factors and intervene early in the natural course of the disease so that intervention can be provided [7]. Various risk calculators that predict CVD risk are available, including the Framingham Risk Score-cardiovascular disease (FRS-CVD), the American College of Cardiology/American Heart Association (ACC/AHA) atherosclerotic cardiovascular disease (ASCVD), QRISK® (current version = QRISK®3), the Joint British Societies Calculator-3 (JBS3), and the WHO/International Society of Hypertension Chart [8-10].

Different researchers have studied the above-mentioned tools in various populations worldwide. At present, no appropriate CVD risk assessment tool concerning the Indian population is available. However, some studies have compared the use of the above-mentioned tools in the Indian population. In a study by Bansal et al., JBS3 and Framingham Risk Score were able to identify those at risk of CVD in patients without the disease [9]. Similarly, Kanjilal et al. studied the various CVD risk calculators available at that time and found them to underestimate CVD risk in the Asian-Indian population [7]. Garg et al. comparatively studied the risk calculators and concluded that FRS-CVD and QRISK®2 performed best in the Indian population in terms of risk assessment and statin eligibility, respectively [8]. QRISK®3, which is an updated and validated version of the QRISK®2 risk calculator with additional parameters for CVD risk prediction, is currently available online [10].

Statin therapy is considered an important preventive measure and has been recommended for patients who are at high risk of CVD [11]. The ACC/AHA-recommended threshold for initiating statin therapy is 7.5% 10-year ASCVD risk. Regarding the National Institute for Health and Care Excellence (NICE) guidelines, the previous cut-off of ≥20% was reduced to ≥10% in 2014 to expand the coverage of statin therapy [12]. In Indian clinical settings, ASCVD score is commonly used as a tool to determine statin eligibility. However, in a study by Garg et al., the ACC/AHA-ASCVD score underestimated the risk of CVD [8].

Considering the above discussion, it is essential to have a reliable and valid tool to predict CVD risk in the Indian population. QRISK®3 has not been evaluated in the Indian population for CVD risk prediction, as we could not find any relevant study after an extensive literature search in PubMed with the key terms “QRISK3” and “QRISK3 AND Indian population.” Therefore, we decided to evaluate this tool in the Indian setting, a tertiary care teaching hospital in central India, to predict 10-year CVD risk in diabetic patients of Indian ethnicity and to categorize them into over-users (i.e., patients who had a 10-year CVD risk of <10% but were receiving statins) and under-users (i.e., patients who had a 10-year CVD risk of ≥10% but were not receiving statins).

## Materials and methods

We conducted a two-month cross-sectional observational study in the outpatient department (OPD) of general medicine at our tertiary care center in Raipur, Chhattisgarh, India. After obtaining approval from the center’s institutional ethics committee, we used convenience sampling to recruit patients for the study. We obtained written informed consent from each patient who was eligible as per the study’s inclusion and exclusion criteria.

Inclusion and exclusion criteria

The inclusion criteria were as follows: diagnosed cases of diabetic patients (both type 1 and type 2), 25-84 years of age, any gender, attending the medicine OPD/diabetic clinic, and consented to participate in the study. The exclusion criteria were as follows: history of CVD (e.g., stroke, transient ischemic attack, myocardial infarction, and angina), and those who were critically ill.

Demographics information

We collected demographic details and relevant health data through a pre-validated structured questionnaire and from available health records. The paper-based prescriptions were provided by the patients. We recorded medication data from these prescriptions, and we screened patients for statin use. We also recorded body mass indices (BMI) and current systolic blood pressure (SBP). Thus, we recorded the parameters required by the QRISK®3 calculator for CVD risk calculation (Appendix).

The above-mentioned parameters were entered into the QRISK®3 online risk tool to calculate the 10-year CVD risk for each patient [13]. The patients were stratified as “over-users” and “under-users” based on their statin use status and 10-year CVD risk, with a cut-off criterion of ≥10%.

Statistical analysis

We expressed categorical data as counts and percentages, and we expressed continuous data as means ± standard deviations. We analyzed the categorical data using Fisher’s exact test. We used Pearson’s correlation coefficient (r) to assess the relationship between various risk factors and 10-year CVD risk scores. A p-value of <0.05 was considered statistically significant.

## Results

We recruited a total of 134 diabetic patients during the study period. A total of 91 patients had a 10-year CVD risk of ≥10% (67.91%, eligible for statin therapy), and 43 patients had a 10-year CVD risk of <10% (32.09%, not eligible for statin therapy). The demographic and clinical details of the study participants are presented in Table 1.

**Table 1:** Demographic and clinical details of the study participants

Variable	n (%)
Gender	
Male	84 (62.69)
Female	50 (37.31)
Age group (in years)	
<40	13 (9.70)
≥40	121 (90.30)
Age in years (mean ± SD)	54.02 ± 11.02
Duration of diabetes	
<1 year	11 (8.21)
≥1 year	123 (91.79)
Smoking status	
Yes	7 (5.22)
No	105 (78.36)
Ex-smoker	22 (16.42)
Alcohol consumption	
Yes	27 (20.15)
No	107 (79.85)
On blood pressure treatment	
Yes	66 (49.25%)
No	68 (51.75%)
Body mass index (in kg/m^2^)	
<18.5	7 (5.22)
18.5-24.9	81 (60.45)
25-29.9	30 (22.39)
>30	16 (11.94)
Education	
Std.	7 (5.22)
Std. 5-10	48 (35.82)
Std. 11-12	25 (18.66)
Graduate	37 (27.61)
Postgraduate	17 (12.69)
Average duration of statin use	26 months

**Figure 1:**
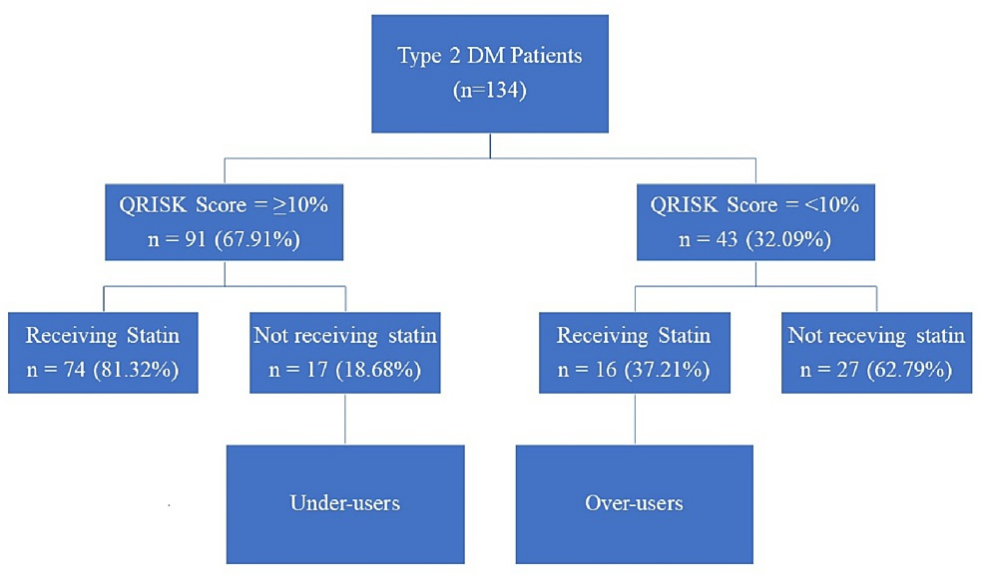
Stratification of statin users as “over-users” and “under-users” DM: diabetes mellitus.

Of the 91 patients with a QRISK score of ≥10%, 74 were on statin therapy, whereas 17 were under-users (i.e., not receiving any statin). Of the 43 patients with a QRISK score of <10%, 16 were over-users (i.e., on statin therapy), whereas 27 were not receiving any statin (Figure 1). The characteristics of the under-users and over-users are depicted in Table 2.

**Table 2:** Characteristics of statin "under-users" and ‘"over-users"

Variables	Over-users (n = 16)	Under-users (n = 17)
Gender		
Male	3	14
Female	13	3
Age group (in years)		
<40	3	0
≥40	13	17
Smoking status		
Yes	0	2
No	15	11
Ex-smoker	3	4
Alcohol consumption		
Yes	0	2
No	16	15
On blood pressure treatment		
Yes	4	9
No	12	8
History of angina or heart attack in first-degree relatives		
Yes	1	1
No	15	16
Average 10-year CVD risk (QRISK score)	6.7%	23.54%
Average duration of statin use (in months)	7 months (vs. 26 months overall)	Not applicable

**Figure 2:**
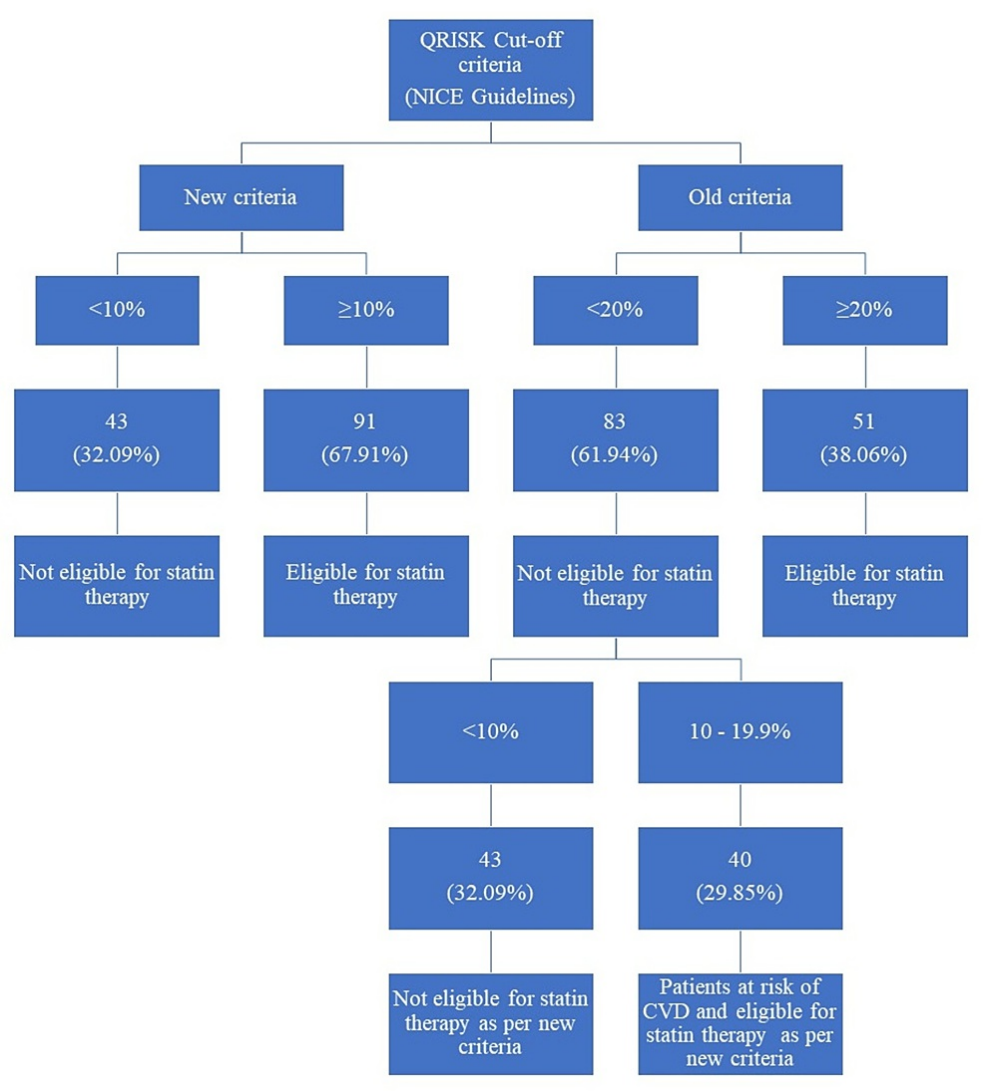
Statin eligibility based on the QRISK score – new versus old cutoff criteria NICE: National Institute for Health and Care Excellence; CVD: cardiovascular disease.

The frequencies of atorvastatin and rosuvastatin prescription were 71.11% and 28.89%, respectively. Statin eligibility was determined by applying new and old cut-off criteria (Figure 2).

Age (p < 0.001), duration of diabetes (p = 0.0011), and current SBP (p < 0.0001) showed a positive correlation with the QRISK score. Waist-to-hip ratio (p = 0.0071) and waist circumference (p = 0.0007) showed a positive correlation with the QRISK score among male patients only. BMI, cholesterol/HDL ratio, non-high-density lipoprotein cholesterol (non-HDL-C), and a standard deviation of two or more in the current SBP showed a weak correlation with the QRISK score (Table 3).

**Table 3:** Correlation of different variables and QRISK score * n = 46; ** n = 101. HDL: high-density lipoprotein; HDL-C: high-density lipoprotein cholesterol; SBP: systolic blood pressure.

Variables	Pearson correlation coefficient (r) – (95% CI)	p-value
Age	0.81 (0.74 – 0.86)	<0.0001
Duration of diabetes	0.27 (0.21 – 0.43)	0.0011
Body mass index	0.09 (-0.07 – 0.26)	0.2529
Waist-to-hip ratio		
Male	0.29 (0.08 – 0.47)	0.0071
Female	0.23 (-0.05 – 0.47)	0.1108
Waist circumference		
Male	0.36 (0.16 – 0.53)	0.0007
Female	0.23 (-0.04 – 0.48)	0.0948
Cholesterol/HDL ratio*	0.26 (-0.03 – 0.51)	0.0878
Non-HDLC*	0.08 (-0.20 – 0.37)	0.5550
Recent SBP (day of visit)	0.48 (0.33 – 0.60)	<0.0001
Standard deviation of two or more recent SBP**	0.18 (-0.01 – 0.36)	0.0756

QRISK score was ≥10% in 58 out of 134 patients receiving blood pressure treatment, which represented a statistically significant correlation (p < 0.0001). Of ex-smokers, 14.94% (20/134) had a QRISK score of ≥10%. A QRISK score of ≥10% was observed in 12 (8.95%) patients who had a history of angina in their first-degree relatives (Table 4).

**Table 4:** Association between angina in first-degree relatives, patients on blood pressure treatment, smoking status, and QRISK score

Parameters	QRISK score		p-value
	<10%	≥10%	
Angina in first-degree relatives			
Yes	1 (0.75%)	12 (8.95%)	0.0607
No	42 (31.34%)	79 (58.96%)	
Patients on blood pressure treatment			
Yes	8 (5.97%)	58 (43.28%)	<0.0001
No	35 (26.12%)	33 (24.63%)	
Smoking status			
Smoker	2 (1.49%)	5 (3.73%)	0.0367
Non-smoker	39 (29.10%)	66 (49.25%)	
Ex-smoker	2 (1.49%)	20 (14.94%)	

## Discussion

In our study, 10-year CVD risk was ≥10% in 91 patients. Therefore, the prescription of statins was justifiable in statin under-users. However, in statin over-users (i.e., CVD risk <10% but receiving statins), statin therapy was not needed. Previous studies have reported similar results, with 24.3% of patients having a CVD risk of <10% prescribed statins [14]. In statin over-users, individual risk factors such as blood pressure treatment, smoking, and alcohol use were less frequent or absent, most likely leading to CVD scores of <10%. The average duration of statin use in these over-users was seven months compared to 26 months in overall users. This result indicates that in over-users, despite less frequent or absent individual risk factors and a CVD risk of <10%, statins were prescribed inappropriately.

The majority of patients were aged ≥40 years (90.30%), with an average age of 54.02 years. In this study, as age increased, CVD risk also increased, and this positive correlation had high statistical significance. This result agreed with previous studies [15].

Over-use of statins is concerning for two reasons: increased risk of adverse effects and increased cost of treatment. Although statistically insignificant, the common adverse effects associated with statin use (e.g., peripheral neuropathy and myopathy) were observed more frequently in stain over-users compared to under-users. However, the diabetic patient population and advanced age could be potential confounders for these findings.

The American Heart Association (AHA) guidelines recommend moderate-intensity statin therapy in all diabetic patients in the 40-75 years age group, and high-intensity statin therapy in those with an AHA-ASCVD risk of ≥7.5%. There are no defined criteria for patient age groups of <40 years and >75 years, which suggests that statins should be prescribed based on the clinical judgment of the physician [16]. DM is itself a risk factor for CVD but cannot serve as a single criterion for statins prescriptions as in the AHA guidelines. Hence, the QRISK score, which is based on several parameters, could be a better criterion for deciding statin eligibility in diabetics as well as in other patients.

In 2014, the NICE guidelines reduced the QRISK cut-off criterion from ≥20% to ≥10% [12]. In our study, we analyzed our data by applying both cut-off criteria. We observed that 29.85% of patients had CVD risk scores of 10-19.9% and were, therefore, ineligible for statin therapy when the old criterion was applied. However, as per the new cut-off, these same patients were eligible for statin treatment. Therefore, it would be inappropriate to miss these potential candidates who are actually at risk of CVD.

We selected the QRISK®3 calculator to predict 10-year CVD risk. This tool has been validated in previous studies [10,17]. In the Indian context, the QRISK®3 calculator was considered the best for statin eligibility, as evident from a study done by Garg et al. [8]. The QRISK®3 calculator is a UK-based risk assessment tool and seems to be best suited for Indians, as compared to US-based risk assessment tools such as AHA-ASCVD. The QRISK tool also has an option to select “Indian” as an ethnicity.

The QRISK tool also covers more extensive parameters than other tools. The inclusion of angina or heart attack in first-degree relatives is important because other observational studies have indicated that premature CVD in a first-degree relative increases the risk of CVD by 50% or more [18,19]. In our study, we also observed that 8.95% of patients with a history of angina or heart attack in their first-degree relatives had QRISK scores of ≥10%.

Although there are some conflicting results about the relationship between the duration of diabetes and CVD risk, the Framingham Heart Study concluded that this relationship represented a positive correlation that was independent of coexisting risk factors [20]. Our results also agreed with these observations, in which the duration of diabetes showed a positive correlation with the QRISK score (p = 0.0011).

Our results also showed a positive correlation between CVD risk and waist circumference and waist-to-hip ratio, which was statistically significant in men. The reason for the weak correlation of waist circumference in women could not be ascertained, but it may be attributable to a smaller number of female participants (50 women vs. 84 men). The literature supports the idea that waist circumference and waist-to-hip ratio are better predictors of CVD risk than BMI [21-23]. However, the QRISK®3 calculator does not include waist circumference or waist-to-hip ratio.

We found that ex-smokers were at higher risk of CVD, as 20 out of 22 (90.91%) patients who were ex-smokers had a QRISK score of ≥10%. In this study, 66 out of 105 (62.85%) non-smokers had a QRISK score of ≥10% (average = 17.15%). The high CVD risk score in this subgroup can be explained by the presence of other risk factors, such as blood pressure treatment (44/66, 66.67%), angina in a first-degree relative, and advanced age (mean age = 53.53 ± 10.86 years), apart from diabetes being present as an independent risk factor. In our study, only 3.73% of the diabetic patients who were smokers had a CVD risk score of ≥10%. This result was as expected and also correlates with previous studies [24]. The association between different risk factors and the QRISK score, which represents the risk of CVD, demonstrated a significant correlation within the Indian patient population.

The QRISK®3 assessment tool could be further improved by adding other risk factors, such as waist circumference, waist-to-hip ratio, and non-HDL-C, as these are now considered better predictors of CVD risk. Due to the relative ease of obtaining waist circumference, its use is favored over waist-to-hip ratio [23]. These factors will not only be helpful in making the risk calculator more accurate but also in decision-making for primary physicians to initiate statins.

The primary limitation of our study was its small sample size. Although the findings from a small group of the population cannot be generalizable, this study showed a positive correlation of various risk factors with CVD risk. Another important limitation was the cross-sectional design of this study. The non-availability of baseline data for some parameters prevented correlation analysis.

## Conclusions

Using the QRISK®3 assessment tool in our study, we determined that DM is an independent risk factor for CVD. Nevertheless, advanced age and the presence of other risk factors increases QRISK score far more than DM alone. To put guidelines into practice, a reliable method of estimating CVD risk is required. A CVD risk score should be determined for all diabetic patients, as it is critical to start statins as a primary prevention of CVD. In our setup, this tool can be used to determine statin eligibility and prevent over-use while also identifying potential candidates for statin initiation and reducing under-use.

## Figures and Tables

**Figure 3:**
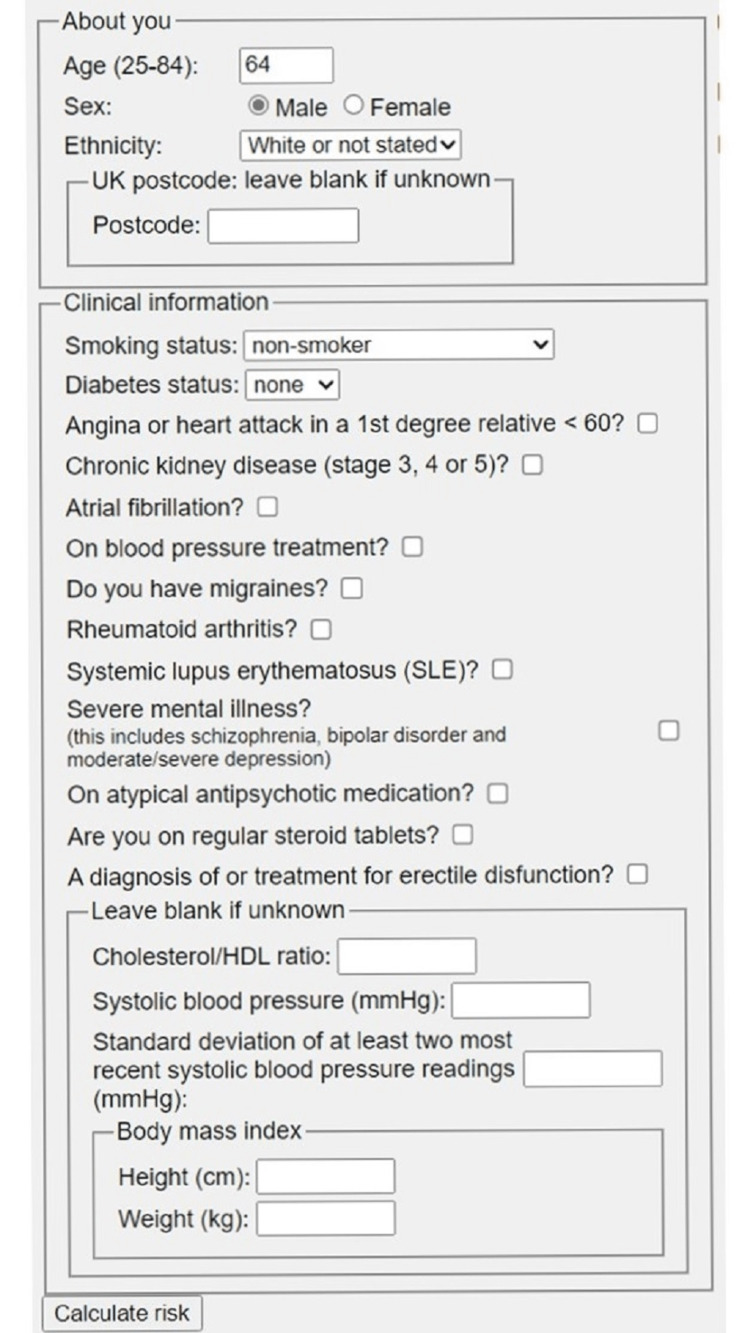
Parameters in QRISK®3 calculator for cardiovascular disease risk calculation
